# What is the current status of care by neuro-otology specialists in Switzerland—A national survey

**DOI:** 10.3389/fneur.2023.1322330

**Published:** 2023-12-07

**Authors:** Andreas Zwergal, Georgios Mantokoudis, Dik Heg, Hassen Kerkeni, Suzie Diener, Roger Kalla, Athanasia Korda, Claudia Candreia, Antje Welge-Lüssen, Alexander A. Tarnutzer

**Affiliations:** ^1^German Center for Vertigo and Balance Disorders, LMU University Hospital, LMU Munich, Munich, Germany; ^2^Department of Neurology, LMU University Hospital, LMU Munich, Munich, Germany; ^3^Department of Otorhinolaryngology, Head and Neck Surgery, Inselspital, Bern University Hospital, University of Bern, Bern, Switzerland; ^4^Clinical Trials Unit Bern, University of Bern, Bern, Switzerland; ^5^Department of Neurology, Inselspital, Bern University Hospital, University of Bern, Bern, Switzerland; ^6^Practice Neurology St. Gallen, St. Gallen, Switzerland; ^7^Department of Otorhinolaryngology, Head and Neck Surgery, Cantonal Hospital Lucerne, Lucerne, Switzerland; ^8^Department of Otorhinolaryngology, Head and Neck Surgery, University Hospital Basel, Basel, Switzerland; ^9^Neurology, Cantonal Hospital of Baden, Baden, Switzerland; ^10^Faculty of Medicine, University of Zurich, Zurich, Switzerland

**Keywords:** vertigo, dizziness, survey, bedside examination, specialists, diagnosis

## Abstract

**Background:**

Vertigo and dizziness are frequent presenting symptoms in the emergency department and in outpatient centers. While the majority of dizzy patients are evaluated by primary care physicians, specialists are often involved in the diagnostic workup. We aimed to gain more insights into the role of specialists in the care of dizzy patients.

**Materials and methods:**

Board-certified neurologists and ear–nose–throat (ENT) physicians working in Switzerland were invited to participate in an online survey. Descriptive statistical analyses were performed, and prospectively defined hypotheses were assessed using correlation analyses.

**Results:**

All 111 participating specialists (neurologists = 62; ENT specialists = 49) were familiar with testing for posterior canal benign paroxysmal positional vertigo (BPPV), and 66% regularly applied provocation maneuvers for suspected lateral canal BPPV. Reposition maneuvers for posterior (99%) and lateral (68%) canals were frequently performed. ENT physicians were familiar with lateral canal BPPV repositioning maneuvers significantly more often than neurologists (84 vs. 56%, *p* ≤ 0.012). Specialists strongly agreed that performing the head impulse test (86%) and looking for deficient eccentric gaze holding (82%) are important. Compared to neurologists, significantly fewer ENT physicians indicated ordering brain MRI in acutely dizzy patients (OR = 0.33 [0.16–0.067], *p* = 0.002) and physical therapy in patients with acute (50 vs. 20%, *p* = 0.005) or episodic/chronic dizziness (78 vs. 50%, *p* = 0.003).

**Conclusion:**

We found substantial differences in the care of dizzy patients by neurologists and ENT physicians. This underlines the need for a standardized, guideline-oriented diagnostic workup and treatment across specialties. Dedicated training for performing lateral canal BPPV repositioning maneuvers should be prioritized for neurologists. Similarly, physical therapy should be considered more often by ENT physicians.

## 1 Introduction

Vertigo and dizziness are among the most frequently reported reasons for seeking medical advice, with a prevalence rate of 8.8 per 1,000 visits in a large ambulatory care survey in the US (1). Although some authors misuse/use these terms interchangeably, the *Bárány* Society (2) has provided precise definitions. Dizziness is the sensation of disturbed or impaired spatial orientation without a false or distorted sense of motion. Vertigo is the sensation of self-motion when no self-motion is occurring or the sensation of distorted self-motion during an otherwise normal head movement. Here, “dizzy patient” and “dizziness” are used in their general sense. While half of all consultations for dizziness were with primary care physicians (PCPs, 51.9%), significant fractions were seen by specialists such as ear–nose–throat (ENT) physicians (13.3%) and neurologists (9.6%) (1). Based on a survey performed by primary care physicians in Switzerland, we identified several significant limitations in the care of dizzy patients (3, 4). This included infrequent use of state-of-the-art neuro-otological bedside examination techniques, a substantial rate of undiagnosed cases reaching 50% in patients with episodic/chronic dizziness, leading to a high referral rate to specialists both in acutely dizzy patients (30%) and patients with episodic or chronic dizziness (50%), and challenges in the interaction between PCPs and specialists such as long waiting periods (4).

In patients assessed by specialized tertiary dizzy clinics, a change in diagnosis can be observed frequently (5–7), as demonstrated in a Swiss academic vertigo center with the fraction of undiagnosed cases decreasing from 70 to 10% (5). Similarly, in a South Korean referral-based dizziness clinic run by neurologists, the fraction of unclear cases was low (5%) (8). While this underlines the importance of highly specialized dizziness clinics in the care of the dizzy patient, such tertiary centers will focus on complex cases and typically have long waiting periods. Consequently, specialists without a sub-specialization in neuro-otology will more likely be involved in the assessment of dizzy patients. Furthermore, based on their individual training, specialists will be more familiar with certain diagnoses leading to dizziness than with others. In a retrospective analysis of a nationwide practice database focusing on ICD-10 codes related to dizziness and vertigo retrieved from 138 ENT practices in Germany, a high rate of unspecific diagnoses (“dizziness and giddiness,” 68%) and a low rate of further referral (e.g., to neurologists, 3.7%) were identified (9). Also, in an emergency department (ED) setting, a surprisingly low rate of agreement on the diagnosis of 39% was found between neurologists and ENT physicians (10). Historically, the wording of identical pathologies was not consistent between ENT specialists and neurologists, which may explain these inconsistencies at least partially.

Similarly, in the ED setting, only a subset of acutely dizzy patients will be seen by specialists. In a secondary Spanish hospital, only 1.7% of all patients presenting with vertigo, gait instability, or dizziness to the ED were evaluated by ENT physicians (11). In contrast, in the ED of a large Swiss academic hospital, with the availability of neurologists and ENT physicians 24/7, substantial fractions of dizzy patients were seen by a neurologist (35.3%) or an ENT physician (11.4%). The resulting rate of vestibular symptoms of unknown origin (14.3%) was below the fraction (20–30%) previously reported (12). This emphasizes the importance of triage and the value of multiple specialized assessments in selected cases with acute dizziness.

We aimed to improve our understanding of the role of specialists in the care of dizzy patients. Therefore a structured questionnaire was designed and sent to both neurologists and ENT physicians. This questionnaire addressed the same aspects as the questionnaire previously used in PCPs (3, 4) and was obtained again in the context of the Swiss healthcare system, but now focusing on the specialists' perspective. Included were assessments of the different diagnostic and therapeutic approaches used by these specialists, including those promoted recently, such as applying the HINTS bedside examination [head impulse, nystagmus, test of skew; (13)] in acutely dizzy patients. We hypothesized that more years of professional experience, the presence of a multi-team or multidisciplinary working place and the specialty would have a significant impact on the approaches used to diagnose and treat dizzy patients and thereby influence the rate of unclear dizzy cases, which would, in turn, need to be referred to other specialist(s).

## 2 Materials and methods

### 2.1 Design of the questionnaire

For this survey-based study, a structured anonymous online questionnaire was designed by the authors (AZ, GM, and AT), targeting board-certified neurologists and ENT physicians working in private practice or in (academic/non-academic) hospitals in Switzerland, referred to as “specialists” in this article. Three main sections were defined to address the pre-specified key aims of the study, reflecting the same overall structure of the survey as previously used for PCPs (3, 4). While in the first section, a detailed picture of current practice when assessing dizzy patients by specialists will be obtained, the second section focuses on identifying limitations faced by the specialist in the diagnostic workup and in the treatment of the dizzy patient. In the third section, the participants will be offered various potential strategies to improve the standard of care for the dizzy patient and the interaction between generalists and specialists. Specifically, the value of different teaching formats was evaluated (see the Appendix in Supplementary material for the full questionnaire). In the questionnaire, key epidemiologic information was also obtained, including the setting of the specialists' working place (location and number of physicians employed), years of professional experience, and educational background. The estimated time needed to fill out the questionnaire was 20–25 min. The questionnaire was available in both German and French, and the translation from German to French was supervised by a native French-speaking expert.

### 2.2 Delivery of the questionnaire and identification of suitable participants

Survey Monkey (Momentive Global Inc., San Mateo, CA, USA) was used for the delivery of this online-only questionnaire, which was sent to suitable physicians based on a database of interested specialists run by healthbook.ch. According to the most recent report of the Swiss Medical Association (FMH) published in 2022, 999 board-certified neurologists and 898 board-certified ENT physicians are currently practicing in Switzerland (14). The target sample size was 150 completed surveys, and we aimed for a proportional representation of participants from all parts of Switzerland. Proportional to the languages spoken in Switzerland, we aimed for 100 questionnaires from specialists living/working in the German-speaking part of Switzerland and 50 questionnaires from specialists located in the French or Italian-speaking part of Switzerland (summarized as “Latin part of Switzerland”). Reimbursement for completion of the questionnaire was provided to each participant to compensate for time and effort spent. Recalls for participation were sent out by email 18 times in total, separately to neurologists and ENT physicians, in the period from February 2022 to September 2022.

### 2.3 Statistical analysis of the questionnaire

In the first step, a descriptive statistical analysis of the questionnaire was made, summarizing epidemiologic data, diagnostic tests performed, and treatments initiated. In a second step, univariable and multivariable statistical analyses were run to validate the pre-specified hypotheses, reflecting the same approach as previously applied when assessing the results from the survey sent to PCPs (3, 4). If the *p*-value was smaller than 0.2 in the univariable analysis, then this variable was also included in the multivariable analysis. Statistical support was provided by DH from the clinical trial unit (CTU) of the University of Bern (Switzerland).

As in the previous studies (3, 4), a series of scores to reflect key aspects of the diagnostic workup (both history taking and bedside testing) were predefined by the authors (AZ, GM, and AT) and were used to correlate with several epidemiologic aspects, including years of professional experience, location of the specialists' office, and reported number of dizzy patients evaluated. These scores were graded based on the extent to which the specialists agreed with a given procedure or the indicated importance of a proposed measure, ranging from, for example, 3 points (very important/fully agreed; 100%) and 2 points (rather important/partially agreed) to 1 point (rather unimportant/partially disagree) and 0 points (not important at all/disagree at all); or if binary, 1 point = agree/used (100%) to 0 points = disagree/not used (0%). All statistical analyses were performed using Stata version 17. Overall scores were derived from the sum of the underlying items and then indexed to 0–100%, or if derived from different scales, first indexed and then averaged. Fractional regressions (odds ratios [OR] with 95% confidence intervals [CI]) are reported for indexed scores; binary dependent variables were analyzed using logistic regression (OR with 95% CI). Descriptive statistics report means with standard deviations (±SD), medians with interquartiles (25–75%), counts with percentages (% of non-missing cases), and sample sizes (number of respondents). See **Figure 3** for a full explanation of each of the scores derived from the respondents' questionnaire items.

## 3 Results

### 3.1 Epidemiologic key aspects of participating specialists

We contacted a total of 959 neurologists and 373 ENT physicians. A total of 111 completed surveys from either board-certified ENT physicians (*n* = 49; return rate = 14.5%) or neurologists (*n* = 62; return rate = 6.5%) were included. Only a minority of participants (36%) were female. Age distribution among the two participating specialties (ENT vs. neurology) was distinct, with a significantly higher fraction of younger (aged 40 years or less) participants in the neurology group than in the ENT physicians' group (40 vs. 8%, *p* < 0.001; for details, see Table 1). While a majority of participating neurologists worked in hospitals (41/62, 66%), this was true only for a minority of ENT physicians (8/49, 16%). For those specialists working in private practice (*n* = 62), offices were located in cities (43/62, 69%), whereas offices in the agglomeration (9%) or rural offices (8%) were less frequent. Participants working in the German part of Switzerland were relatively overrepresented compared to participants from the Latin part of Switzerland (78 vs. 22%).

**Table 1:** Epidemiologic key results of participating specialists.

	***n* (%)**
**Sex**	
Female	40 (36%)
Male	71 (64%)
**Age distribution**	
30–40 years	29 (26%)
41–50 years	33 (30%)
51–60 years	28 (25%)
>60 years	21 (19%)
**Specialty of participating board-certified physicians** ^*^	
ENT physicians	62
Neurologists	49
**Geographical location of participating specialists' office**	
German part of Switzerland	87 (78%)
Latin (i.e., French/Italian-speaking) part of Switzerland	24 (22%)
**Setting of specialists' office**	
**Private practice**	
Private practice in the countryside	9 (8%)
Private practice in the agglomeration	10 (9%)
Private practice in the city	43 (39%)
All	62 (56%)
**Hospital**	
University hospital	24 (22%)
Non-university hospital	25 (23%)
All	49 (44%)
**Number of specialists working at the participant's location**	
1	28 (25%)
2–4	42 (38%)
5–8	15 (14%)
>8	26 (23%)
**Years of professional experience (after finishing their studies)** ^*^	
Participating ENT physicians	26.9 ± 9.4 years (*n* = 49)
Participating neurologists	17.9 ± 9.5 years (*n* = 62)
**Number of patients seen per day (average** ±**1** ***SD***)^*^	
Participating ENT physicians	21.0 ± 7.6 (*n* = 49)
Participating neurologists	9.8 ± 3.4 (*n* = 62)
**Time spent per consultation (minutes, average** ±**1** ***SD***)^*^	
Participating ENT physicians	20.5 ± 5.9 (*n* = 49)
Participating neurologists	27.4 ± 5.3 (*n* = 62)
**Number of patients seen with a leading symptom of**	
**vertigo or dizziness per month (average** ±**1** ***SD*****)**	
Participating ENT physicians	20.7 ± 19.0 (*n* = 49)
**Time spent per consultation for patients presenting**	
**with vertigo or dizziness**	
**As much time as for other patients on average** ^*^	
Participating ENT physicians	2 (4%)
Participating neurologists	33 (53%)
**More time as for the average patient** ^*^	
Participating ENT physicians	47 (96%)
Participating neurologists	29 (47%)

^*^There was a significant difference between ENT physicians and neurologists (Fisher's exact test, *p* < 0.001).

While the majority of ENT physicians worked alone (37%) or in small offices (2–4 physicians, 49%), most neurologists worked in larger offices (five or more physicians, 55%). The average (±1 *SD*) number of years of working experience of participating specialists was 21.9 ± 10.4 years, with ENT physicians reporting significantly higher values than neurologists (26.9 ± 9.4 vs. 17.9 ± 9.5 years, *p* < 0.001). On average (±1 *SD*), participating specialists saw 14.7 ± 7.9 patients per day, spending 24.4 ± 6.5 min per patient. Importantly, ENT physicians reported seeing a significantly higher number of patients per day, resulting in lower consultation times (for details, see Table 1). Over the period of a month, the number of patients seen with a leading symptom of dizziness averaged 19.3 (±15.9, 1 *SD*, range: 0–100 patients). Whereas, 96% of participating ENT physicians indicated that they spend more time on average with a patient presenting with dizziness than with a patient reporting other chief complaints, this number was significantly (*p* < 0.001) lower for neurologists (47%).

### 3.2 History taking in patients presenting with vertigo or dizziness

**Figure 1:**
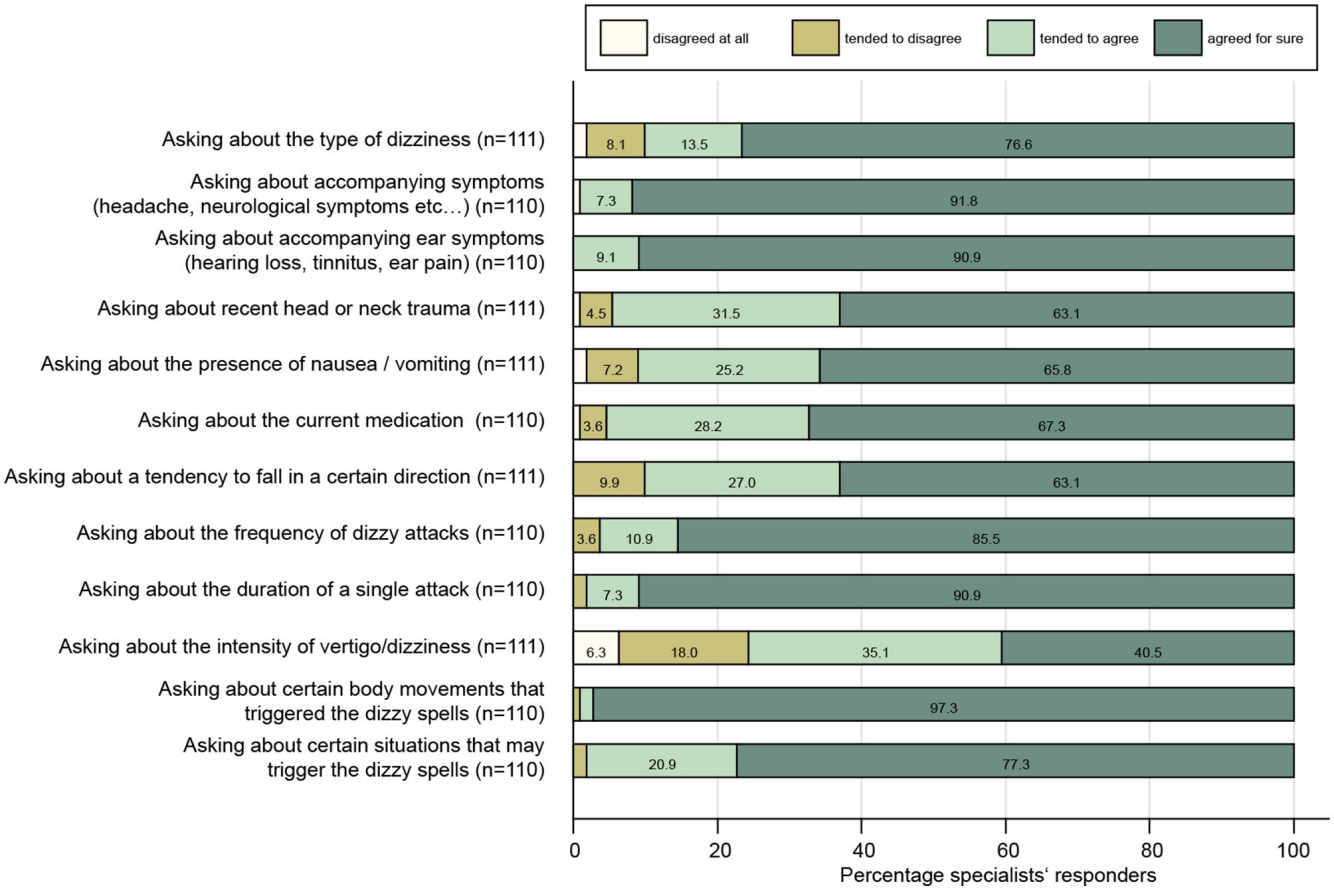
Response patterns of participating specialists are shown for a series of questions when taking the dizzy patient's history. For each question, the percentage of specialists and the level of agreement they indicated (ranging from “disagree at all” to “agreed for sure”) are illustrated. For each question, the number (*n*) of valid replies is provided in brackets.

With regard to history taking in the dizzy patient, participating specialists agreed for sure or tended to agree to all proposed questions with rates of 76–98% (see Figure 1). Highest rates for strong agreement received the questions asking about body movements that triggered dizzy spells (97%), asking about accompanying (ear) symptoms (91–92%), and about the duration of a single attack (91%), whereas lowest fractions for strong agreement were found for asking about the intensity of vertigo/dizziness (41%), a recent head/neck trauma (63%), and falls in a certain direction (63%).

### 3.3 Clinical examination of the patient presenting with vertigo or dizziness

#### 3.3.1 Bedside tests performed

**Figure 2:**
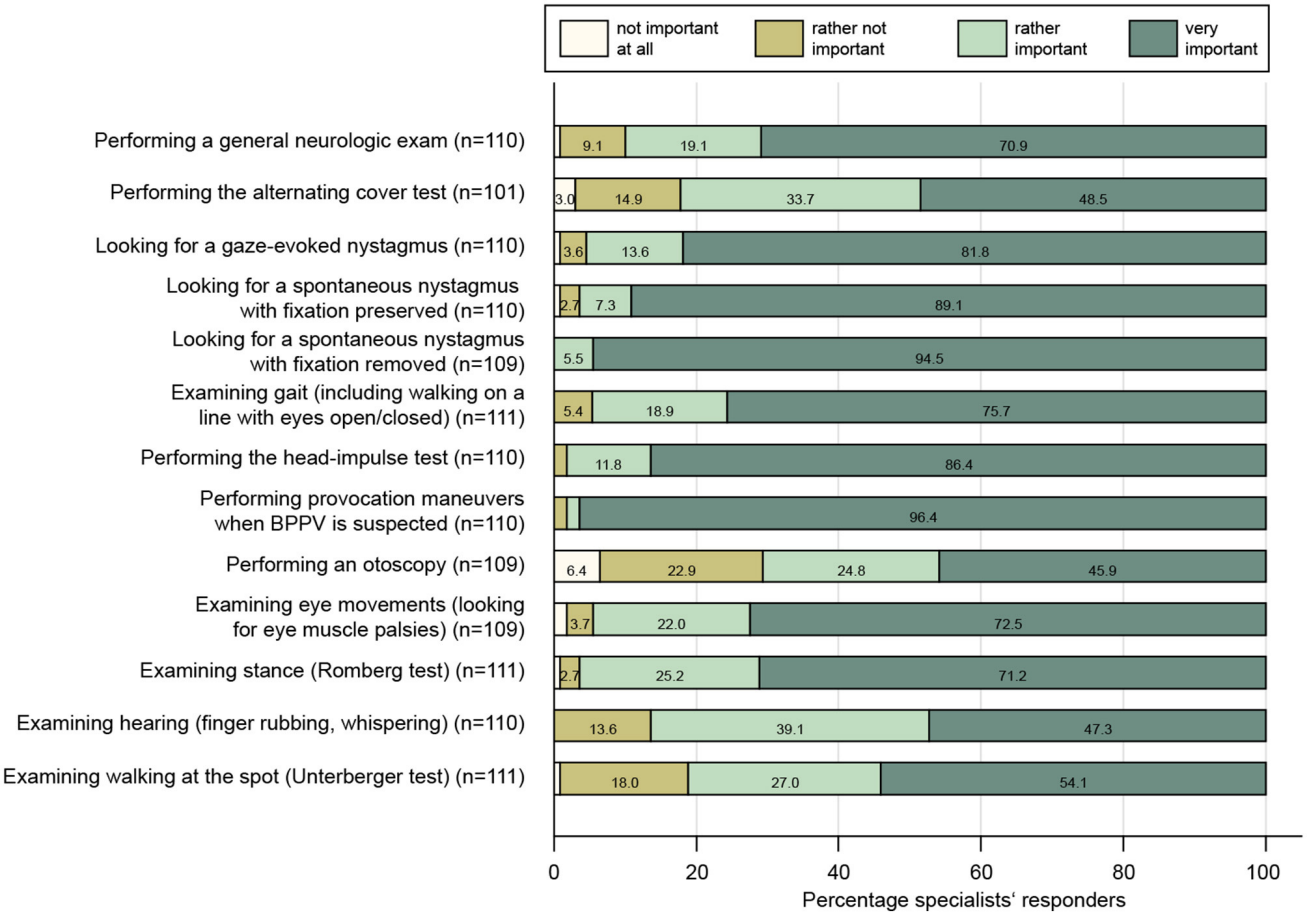
Response patterns of participating specialists are shown for a series of clinical examinations when assessing the dizzy patient. For each question, the percentage of specialists and the level of importance they indicated (ranging from “not important at all” to “very important”) are illustrated. For each question, the number (*n*) of valid replies is provided in brackets.

**Figure 3:**
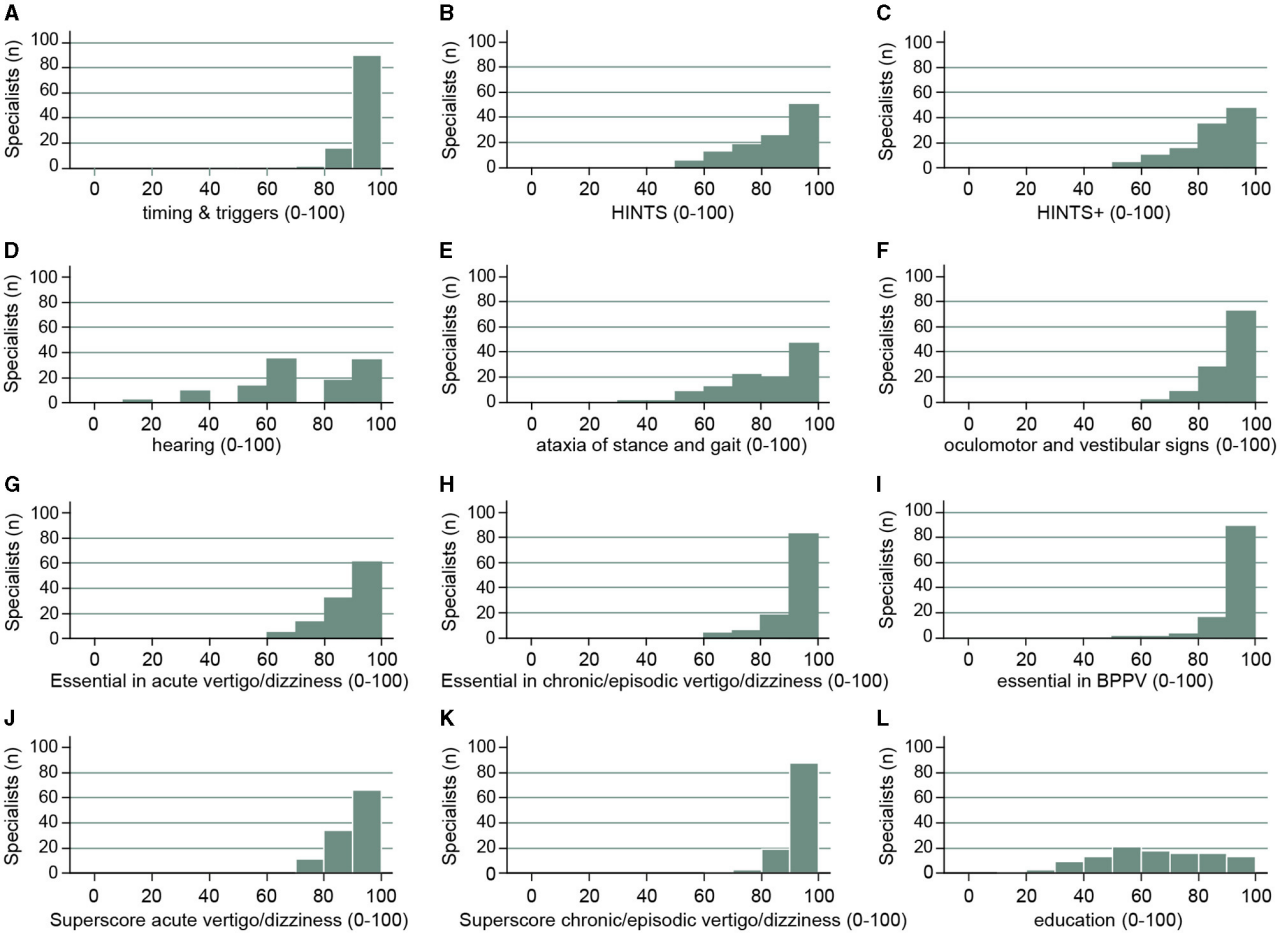
Specialists' (neurologists, ENT) performance for various scores is illustrated. These scores are identical to the ones previously used in the survey sent to PCPs (4). This included the following scores: *timing and triggers* [**A**: asking for the frequency and duration of dizzy spells, triggers (specific body movements/positions, specific situations), accompanying symptoms; 16], *HINTS* (**B**: performing the head impulse test, looking for gaze-evoked nystagmus and for skew deviation), *HINTS*+ (**C**: HINTS plus looking for new-onset unilateral hearing loss), *hearing* (**D**: testing for new-onset hearing loss, performing otoscopy), *ataxia of stance and gait* (**E**: assessment of walking on the line (with/without viewing), Romberg test, Unterberger stepping test), *subtle oculomotor and vestibular signs* (**F**: performing HINTS and testing for spontaneous nystagmus with both fixation preserved and removed), “*essential” in acute vertigo/dizziness* (panel **G**: testing for HINTS+, assessment of walking on the line (with/without viewing), Romberg test and for spontaneous nystagmus with both fixation preserved and removed), “*essential” in episodic/chronic vertigo/dizziness* (**H**: performing provocation maneuvers, the head impulse test, assessments of walking on the line (with/without viewing) and the Romberg test), “*essential” in suspected BPPV* (**I**: asking for timing and triggers and performing provocation maneuvers), *superscore acute vertigo/dizziness* (**J**: essential in acute vertigo/dizziness and timing and triggers), *superscore for episodic/chronic vertigo/dizziness* (**K**: essential in episodic/chronic vertigo/dizziness and timing and triggers), *education* (**L**: analog media (hands-on courses, workshops, national recommendations, practical recommendations) and digital media (smartphone apps, webinars).

When examining the dizzy patient at the bedside, participating specialists agreed for sure or tended to agree to all proposed tests with rates of 71–98% (see Figure 2). The highest rates of approval were found for performing provocation maneuvers when benign paroxysmal positional vertigo (BPPV) was suspected (96%), for looking for a spontaneous nystagmus with fixation removed (95%) or preserved (89%), for performing the head impulse test (86%), and for looking for gaze-evoked nystagmus (82%). In contrast, rates of strong agreement for performing an otoscopy (46%), examining hearing (47%), and performing the alternating cover test (49%) were the lowest. We calculated a set of scores to assess the specialists' familiarity with structured diagnostic algorithms in the dizzy patient based on history-taking and bedside tests (see Figure 3 and its legend for details). While for some scores most specialists performed well, such as timing and triggers (Figure 3A), “essential” in BPPV (Figure 3I), and the “superscore” in episodic/chronic dizziness (Figure 3K), they scored lower in other scores, including HINTS+ (Figures 3B, C), ataxia of stance and gait (Figure 3E), and education (Figure 3L).

In the next step, we evaluated the use of HINTS (i.e., performing the head impulse test, testing for gaze-evoked nystagmus and for skew deviation [see (13)]) or its extension (the HINTS+, including new-onset unilateral hearing loss [see (15)]) in patients with acute prolonged dizziness. Specifically, we assessed the impact of the specialists' age on the calculated HINTS+ composite score (i.e., a score based on the indicated importance of the HINTS+ components by the specialists). Noteworthy, no significant correlations were observed for both the HINTS score (*p* = 0.20) and the HINTS+ score (*p* = 0.66). This was also true when adding testing for spontaneous nystagmus (with and without fixation) to the HINTS bedside examination, again showing no correlation of this “subtle oculomotor and vestibular signs” score with specialists' age (*p* = 0.31). In comparison, interviewed specialists (ENT physicians vs. neurologists) showed significantly different results in several scores. Specifically, ENT physicians scored significantly higher in the “audio-vestibular testing score” (reflecting the availability of quantitative audio-vestibular testing including video-head impulse testing, caloric irrigation, video-oculography, vestibular-evoked myogenic potentials, etc.) (28.6 [14.3; 71.4] vs. 14.3 [0.0; 28.6], *p* < 0.001) and in the “hearing score” (including otoscopy and testing hearing by use of finger rub) (100 [83.3; 100] vs. 66.7 [50; 66.7], *p* < 0.001).

#### 3.3.2 Tools available for the examination

Asked about bedside tools available for the clinical neuro-otological examination, almost all participating specialists indicated that Frenzel's goggles (94%) were available, whereas video Frenzel's goggles were more often used by ENT physicians than by neurologists (69 vs. 11%, *p* < 0.001). Similarly, almost all ENT physicians used an otoscope, whereas rates for neurologists were lower (100 vs. 65%, *p* < 0.001). The opposite was true with regard to the use of a vibration tuning fork (94 vs. 78% [neurologists vs. ENT physicians], *p* = 0.023) and an eye chart (65 vs. 16%, *p* < 0.001).

Overall, quantitative audio-vestibular testing was more frequently used by ENT physicians than by neurologists. Significantly higher rates of ENT physicians than neurologists reported the use of hearing testing, including smartphone-based applications, video-head impulse testing, caloric irrigation, and cervical vestibular-evoked myogenic potentials. In contrast, all other quantitative testing was used by both specialties at similar frequencies (see Table 2 for details).

**Table 2:** Diagnoses most frequently made and diagnostic tools available (ENT, neurology).

**Diagnoses made (in order of decreasing frequency)**	**Ranking (median, interquartile range [25%; 75%])**
Benign paroxysmal positional vertigo	1.0 [1.0; 2.0]
Multifactorial dizziness	4.0 [2.0; 5.0]
Acute unilateral vestibulopathy	4.0 [3.0; 6.0]
Functional dizziness (“phobic vertigo”)	4.5 [3.0; 6.0]
Dizziness/gait imbalance linked to peripheral polyneuropathy	5.0 [3.0; 7.0]
Vestibular migraine	6.0 [4.0; 8.0]
Vertigo or dizziness of unclear origin	6.0 [5.0; 8.0]
Menière's disease	7.0 [5.0; 9.0]
Vertigo or dizziness related to cardiovascular disease	9.0 [7.0; 9.0]
Diagnostic tools available	
175,17497pt	**Neurologists**	**ENT physicians**	**statistical analysis**
**Bedside diagnostic tools**			
Frenzel goggles	57/62 (92%)	47/49 (96%)	*p* =0.46
Video Frenzel goggles	7/62 (11%)	34/49 (69%)	*p* < 0.001
Eye chart	40/62 (65%)	8/49 (16%)	*p* < 0.001
Tuning fork	58/62 (94%)	38/49 (78%)	*p* = 0.023
Otoscope	40/62 (65%)	49/49 (100%)	*p* < 0.001
**Quantitative audio-vestibular testing**			
Pure tone audiometry (including smartphone-based testing)	12/62 (19%)	49/49 (98%)	*p* < 0.001
Video-head impulse test	15/62 (24%)	31/49 (63%)	*p* < 0.001
Video-oculography	11/62 (18%)	16/49 (33%)	*p* = 0.079
Caloric irrigation	13/62 (21%)	46/49 (94%)	*p* < 0.001
Ocular vestibular-evoked myogenic potentials	8/62 (13%)	13/49 (27%)	*p* = 0.089
Cervical vestibular-evoked myogenic potentials	5/62 (8%)	16/49 (33%)	*p* = 0.001
Subjective visual vertical	24/62 (39%)	19/49 (39%)	*p* = 1.00
Posturography	6/62 (10%)	9/49 (18%)	*p* = 0.26
Turntable for diagnosing/treating BPPV	9/62 (15%)	9/49 (18%)	*p* = 0.61
Rotatory chair (testing of OKN, VOR)	7/62 (11%)	4/49 (8%)	*p* = 0.75

BPPV, benign paroxysmal positional vertigo; ENT, ear–nose–throat; OKN, optokinetic nystagmus; VOR, vestibulo-ocular reflex.

#### 3.3.3 Frequency of diagnoses in patients presenting with vertigo or dizziness

Asking to provide a ranking among a selection of nine potential diagnoses in the dizzy patient, “BPPV” was by far the most frequent diagnosis made by specialists, ranking first in 60.4% of participants (median and interquartile range [IQR, 25%−75%]: 1.0 [1.0; 2.0]). The second frequent diagnosis was “multifactorial dizziness” (ranking first place in 10.2% and second place in 17.7% of specialists, with a median ranking of 4.0 [2.0; 5.0]). The diagnosis of “acute unilateral vestibulopathy” followed in third place (ranking first in 4.6% of specialists, second in 19.4%, and third in 20.4%, with a median ranking of 4.0 [3.0; 6.0]; see Table 2). When assessing the rankings made by ENT physicians and neurologists separately, we noted differences in the ranking list. While BPPV was the most frequent diagnosis in both specialties, acute unilateral vestibulopathy and Menière's disease were ranked higher by ENT physicians than by neurologists. In contrast, dizziness and gait imbalance related to peripheral neuropathy were more often diagnosed by neurologists than by ENT physicians (for details, see Supplementary Table 1).

#### 3.3.4 Diagnosis and treatment of BPPV

All specialists were familiar with the provocation maneuver for testing for posterior canal BPPV (i.e., the Hallpike-Dix maneuver, 100%), and a majority of them regularly applied provocation maneuvers for diagnosing lateral canal BPPV [supine roll maneuver (also known as the Pagnini-McClure maneuver)] (66%), whereas a minority only was familiar with the bow and lean test (12%, see Table 3). Asked about reposition maneuvers, 99% of specialists confirmed the use of at least a single repositioning maneuver for posterior canal BPPV, with the Epley maneuver (94%) being more frequently applied than the Semont maneuver (76%). A majority (78/111, 68%) of specialists indicated being familiar with at least one treatment maneuver for lateral canal BPPV, with higher numbers for the Barbecue maneuver (59%) than for the Gufoni maneuver (45%). Overall, ENT physicians were familiar with lateral canal BPPV repositioning maneuvers significantly more often than neurologists [using at least one repositioning maneuver in 84% (41/49 cases) compared to 56% (35/62 cases), ENT physicians vs. neurologists], as illustrated in detail in Table 3.

**Table 3:** Treatment strategies in dizzy patients.

	**Overall**	**Neurologists**	**ENT physicians**	**Statistics**
**Treatment options considered in patients with acute dizziness/vertigo**	**Fractions (%, median [IQR])**			
Physical therapy	25% [10.0%; 60.0%]	50% [18.8%; 71.3%]	20% [8.5%; 40.0%]	*p* = 0.005
Antiemetic drugs	27% [10.0%; 50.0%]	28% [8.8%; 50.0%]	27% [10.0%; 63.0%]	*p* = 0.65
Anti-vertiginous drugs	20% [5.0%; 50.0%]	10% [0.8%; 37.8%]	40% [15.0%; 70.0%]	*p* < 0.001
**Treatment options considered in patients with episodic or chronic dizziness/vertigo**	**Fractions (%, median [IQR])**			
Physical therapy	60% [35%; 80%]	78% [50%; 95%]	50% [30%; 71.5%]	*p* = 0.003
Antiemetic drugs	5% [0%; 13%]	5% [0%; 15%]	5% [0%; 11%]	*p* = 0.85
Anti-vertiginous drugs	20% [1%; 50%]	5% [0%; 21.3%]	40% [16%; 60%]	*p* < 0.001
**Anti-vertiginous drugs regularly prescribed in dizzy patients**				
Betahistine	90/111 (81%)	45/62 (73%)	45/49 (92%)	*p* = 0.014
Cinnarizine + dimenhydrinate	52/111 (47%)	18/62 (29%)	34/49 (69%)	*p* < 0.001
Steroids	51/111 (46%)	17/62 (27%)	34/49 (69%)	*p* < 0.001
Ginkgo biloba	45/111 (41%)	14/62 (23%)	31/49 (63%)	*p* < 0.001
Flunarizine	25/111 (23%)	16/62 (26%)	9/49 (18%)	*p* = 0.37
**Diagnostic and therapeutic procedures in patients with (suspected) BPPV**				
**Diagnostic maneuvers in BPPV applied**	**Fractions (%)**			
Hallpike-Dix maneuver	111/111 (100%)	62/62 (100%)	49/49 (100%)	*p* = 1.00
Supine roll maneuver (90° Barbecue maneuver)	73/111 (66%)	39/62 (63%)	34/49 (69%)	*p* = 0.55
Inverse Hallpike maneuver^*^	21/111 (19%)	11/62 (18%)	10/49 (20%)	*p* = 0.81
Bow and lean test	13/111 (12%)	4/62 (6%)	9/49 (18%)	*p* = 0.07
**Therapeutic maneuvers in BPPV performed**				
Epley maneuver	104/111 (94%)	58/62 (94%)	46/49 (94%)	*p* = 1.00
Semont maneuver	84/111 (76%)	46/62 (74%)	38/49 (78%)	*p* = 0.82
Gufoni maneuver	50/111 (45%)	21/62 (34%)	29/49 (59%)	*p* = 0.012
Barbecue maneuver	66/111 (59%)	28/62 (45%)	38/49 (78%)	*p* = 0.001

^*^Looking for anterior canal BPPV. BPPV, benign paroxysmal positional vertigo; IQR, interquartile range.

Among participating specialists, a large majority confirmed that they provided verbal instructions (73%/20%; always/often) or brochures/drawings (59%/22%) for self-repositioning maneuvers to patients with diagnosed BPPV. Referring to web-based teaching videos for self-repositioning maneuvers was always/often true for 25–32% of participating specialists. A minority of specialists only indicated that they always or often prescribed anti-vertiginous drugs (2%/6%) or antiemetic drugs (2%/19%) to patients with suspected BPPV. Few specialists indicated that they always (9%) or often (16%) prescribed vitamin D to patients with recurrent BPPV.

### 3.4 Prescribed treatment

Among the proposed treatment options for dizzy patients, a minority of specialists selected physical therapy (25%), antiemetic drugs (27%), or anti-vertiginous drugs (20%) for acutely dizzy patients. Importantly, neurologists indicated prescribing physical therapy significantly more often than ENT physicians (50% [18.8; 71.3%] vs. 20% [8.5; 40%], *p* = 0.005). In contrast, ENT physicians significantly more often indicated recommending anti-vertiginous drugs in acutely dizzy patients (40% [15; 70%] vs. 10% [0.8; 37.8%], *p* < 0.001). In patients with episodic/chronic dizziness, the rates for prescribing physical therapy were higher (60%), whereas anti-vertiginous drugs and antiemetic drugs were prescribed less frequently than in acutely dizzy patients (see Table 3 for details). Noteworthy, neurologists significantly more often recommended physical therapy (78% [50; 95%] vs. 50% [30; 71.5%], *p* = 0.003), whereas ENT physicians prescribed anti-vertiginous drugs significantly more frequently (40% [16; 60%] vs. 5% [0; 21.3%], *p* < 0.001). Betahistine and cinnarizine–dimenhydrinate were the most commonly recommended anti-vertiginous drugs for dizzy patients (acute or episodic/chronic). Overall, ENT physicians indicated prescriptions of all enlisted anti-vertiginous drugs (except for flunarizine) more often than neurologists (see Table 3 for details).

### 3.5 Managing suspected acute unilateral vestibulopathy

A small majority of participating neurologists indicated that it was always (35%) or often (19%) true that patients with a diagnosis of (suspected) acute unilateral vestibulopathy are sent for further evaluation or treatment to an ENT specialist or another neurologist. In contrast, agreement rates for referral to a neurologist or another ENT physician were much lower (always true = 5%, often true = 0%) among ENT physicians.

A smaller fraction of specialists indicated that they would refer such patients always (4%) or often (10%) to an emergency physician. Asked about preferences with regard to a referral to radiology for brain imaging, only a minority of specialists agreed that they would always or often order a computed tomography (CT) scan (always: 1%, often: 4%) or magnetic resonance imaging (MRI) (always: 5%, often: 19%) in patients with (suspected) acute unilateral vestibulopathy.

When performing a univariable logistic regression analysis with regard to the odds for ordering a brain MRI in patients with suspected acute unilateral vestibulopathy, only the specialty (ENT vs. neurology) showed a significant impact. Specifically, ENT physicians significantly less often ordered an MRI than neurologists (OR = 0.33 [0.16–0.67], *p* = 0.002). This was confirmed in a multivariable analysis (see Supplementary Table 2).

With regard to treatment strategies in patients with acute unilateral vestibulopathy, a majority of specialists indicated that they would initiate symptomatic treatment with antiemetics (24%/52%; always/often true) or anti-vertiginous drugs (17%/38%). Prescribing steroids was considered by a majority of specialists (with no significant differences in frequency between neurologists and ENT physicians) (47%/34%), whereas antiviral drugs were proposed by a minority of participating specialists only (2%/14%). Remarkably, we did not identify any significant correlations between the monthly total number of dizzy patients seen by the specialist or the fraction of acutely dizzy patients receiving no specific diagnosis after initial assessment and the frequency of prescribing physical therapy, antiemetics, or anti-vertiginous drugs (see Supplementary Table 3).

### 3.6 Managing episodic/chronic vertigo or dizziness

A small majority of participating specialists indicated that they would always (46%) or often (20%) perform provocation maneuvers for possible BPPV in patients presenting with episodic or chronic dizziness. With regard to treatment strategies for patients with episodic or chronic dizziness, 60% of specialists indicated that they would prescribe physical therapy for balance training, whereas rates for pharmaceutical treatment were much lower (see Table 3). When asked how often they would do so, over 90% indicated frequent prescription of physical therapy (45%/46%; always true/often true), whereas a minority would initiate a symptomatic treatment with anti-vertiginous drugs (5%/33%) or would prescribe antiemetic drugs (3%/10%). Only a few specialists indicated that they would take no action but only follow up on these patients (0%/13%; always/often true). We did not identify any significant correlations between the number of dizzy patients seen per month by the specialist or the fraction of patients with episodic/chronic dizziness receiving no specific diagnosis after initial assessment and the frequency of prescribing physical therapy, antiemetics, or anti-vertiginous drugs (see Supplementary Table 3).

## 4 Discussion

The primary purpose of this online survey (representing about 6.5% of all board-certified neurologists and 14.5% of all board-certified ENT physicians working in Switzerland) was to gain more knowledge about the current exposure of ENT physicians and neurologists to dizzy patients and their application of diagnostic and therapeutic procedures. We have previously addressed the same items in primary care physicians (PCPs) (3, 4). Gaining more knowledge about the current state of care of the dizzy patient in Switzerland from the specialists' perspective is an important prerequisite to identifying and prioritizing current limitations and needs.

Noteworthy, the two specialties evaluated by the questionnaire have different professional profiles, which is important to consider when interpreting the findings. The ENT physicians included reported significantly more working experience than the neurologists (26.9 ± 9.4 vs. 17.9 ± 9.5 years, *p* < 0.001), worked in private practice in the majority (84%, whereas neurologists worked in hospitals in 66% of cases), and spent significantly less time on the average patient than the neurologists (20.5 ± 5.9 min vs. 27.4 ± 5.3 min, *p* < 0.001). Thus, seeing dizzy patients disrupted their schedule much more than neurologists. However, the number of dizzy patients seen by both ENT physicians and neurologists on a monthly basis was very similar, on average approximately 20.

Both the diagnostic approach and the treatment strategy strongly depended on the suspected cause of dizziness and the specialists' training background. For example, neurologists ordered brain imaging significantly more often than ENT physicians. Whereas, neurologists indicated prescribing physical therapy most often both for patients with acute and episodic/chronic dizziness and rarely (in 10% of cases or less) recommended the use of anti-vertiginous drugs, ENT physicians preferred prescribing anti-vertiginous drugs over physical therapy in acutely dizzy patients. Similarly, for patients with episodic/chronic dizziness, ENT physicians prescribed anti-vertiginous drugs almost at the same rate as physical therapy (40 vs. 50%).

### 4.1 History taking, clinical examination, and quantitative audio-vestibular testing

We found that specialists are very familiar with the concept of dizzy spells being triggered by certain body movements, whereas asking for situational triggers (as seen in functional dizziness) is considered somewhat less important. Almost all participating specialists considered questions about the frequency and duration of attacks as important. This is consistent with modern concepts of addressing patients' complaints, focusing on timing and triggers [TiTrATE approach; (16)], demonstrating a clearly different picture as previously noted in PCSs in the companion survey (4). However, asking for a recent head or neck trauma (full approval: 63%) or about current medication (full approval: 67%) was considered important less often, indicating limitations in current history-taking strategies by specialists.

We found that specialists are well aware of the importance of performing provocation maneuvers in patients with suspected BPPV, looking for spontaneous nystagmus (with fixation removed) in dizzy patients, obtaining the head impulse test, and looking for gaze-evoked nystagmus, whereas performing a general neurologic examination and an analysis of stance and gait were considered somewhat less important. Looking for a vertical skew in the dizzy patient was considered very important in 49% of specialists only. Similarly, assessment of hearing (by finger rubbing) was considered very important in only 47%. Thus, while testing for subtle oculomotor findings was considered very important by a larger fraction of participating specialists than by PCPs [as assessed in a previous study from our group (3)], bedside evaluation for a skew deviation and for acute (unilateral) hearing loss, which are part of the HINTS+ examination that offers high diagnostic accuracy for central causes in acutely dizzy patients (15), was not retrieved as thoroughly. There should be more emphasis on testing for a skew deviation and for new-onset hearing loss by specialists when dealing with acutely dizzy patients. In patients with episodic or chronic vertigo/dizziness, however, these clinical findings are less important, thus a selection bias (preferentially seeing patients with episodic/chronic complaints) may have affected the specialist's preference for testing for skew deviation and hearing loss.

ENT physicians had access to quantitative audio-vestibular testing significantly (*p* ≤ 0.011) more often than neurologists; this included obtaining a video-head impulse test (63 vs. 24%), a hearing test (98 vs. 19%), caloric irrigation (94 vs. 21%), and cervical VEMPs (33 vs. 8%). This will allow ENT physicians to obtain a more detailed diagnostic workup and to identify conditions such as bilateral vestibulopathy that may otherwise remain undetected. However, we did not observe any significant differences in the reported rates of patients who received no specific diagnosis after a diagnostic workup had been performed comparing ENT physicians vs. neurologists. Likely, based on the working diagnosis of the referring physician, ENT physicians, and neurologists will assess distinct patient populations (i.e., those patients with suggestive peripheral vestibular deficits will be referred to ENT physicians more likely, whereas those patients with central findings will be sent to neurologists) as this has been previously shown in a German survey (17).

### 4.2 Diagnostic workup and treatment strategies in acutely dizzy patients

As part of the diagnostic workup of the acutely dizzy patient, brain imaging (CT 5%, MRI 24%) was ordered always (or often) by only a minority of the specialists. Noteworthy, board-certified ENT physicians significantly less often ordered an MRI compared to neurologists (OR = 0.33 [0.16–0.67], *p* = 0.002), which could be related to the distinct spectrum of differential diagnoses exposed to most often. In line with our findings, in a previous study, neurologists more often ordered imaging than ENT physicians for patients with vestibular diagnoses (16.5 vs. 4.7%) (1). In the context of suspected acute unilateral vestibulopathy, specialists indicated starting a steroid treatment always or often in 81% of cases (with no significant differences in prescription behavior between neurologists and ENT physicians), which is substantially higher than the 46% reported by PCPs (3). Apparently, specialists working in Switzerland follow closely the recent guidelines promoting steroid treatment in patients with acute unilateral vestibulopathy [see, e.g., the guideline from the German ENT society (18)]. Whereas, in acutely dizzy patients (not restricted to acute unilateral vestibulopathy), antiemetics were frequently prescribed by specialists (76%), and anti-vertiginous drugs were considered always (or often) only by about half of specialists (51%). In comparison to PCPs, rates for prescribing antiemetics and anti-vertiginous drugs were almost identical. As in PCPs, it will be important to restrict the use of vestibular suppressants to the acute stage as they may inhibit central vestibular compensation when taken for more than 2–3 days and are thus largely inappropriate (19). Noteworthy, ENT physicians indicated the use of anti-vertiginous drugs in acutely dizzy patients significantly more often than neurologists (40 vs. 10%); therefore, it will be especially important to increase awareness of this limitation among ENT physicians.

With regard to non-pharmaceutical treatment strategies in acutely dizzy patients, in general, this was considered substantially more often by neurologists than by ENT physicians (50 vs. 20%). The level of awareness of the concept of vestibular rehabilitation seems to differ between the two specialist groups included here (20). This observation is consistent with a previous study, reporting higher referral rates to physical therapy for BPPV and other vestibular disorders in neurologists than ENT physicians (19.3 vs. 0.8%) (1). We conclude that to promote physical therapy in acutely dizzy patients, reaching out to ENT physicians must be prioritized.

### 4.3 Diagnostic workup and treatment strategies in patients with episodic or chronic dizziness including benign paroxysmal positional vertigo

Specialists' knowledge about diagnosing and treating BPPV depended on the canal affected and their training background. While virtually all specialists indicated being very familiar with performing diagnostic (100%) and therapeutic (99%) maneuvers for posterior canal BPPV, rates for diagnostic (68%) and therapeutic (66%) procedures performed for suspected lateral canal BPPV were substantially lower. Importantly, ENT physicians indicated the use of lateral canal repositioning maneuvers significantly more often than neurologists; this was true both for the Gufoni maneuver (34 vs. 59%) and the Barbecue maneuver (45 vs. 78%). This indicates a gap of knowledge that was substantially more prevalent among neurologists than ENT physicians and that must be addressed. We conclude that training for lateral canal BPPV repositioning maneuvers should be prioritized for neurologists. In comparison with results from a survey distributed among Lithuanian physicians, who reported limited use of provocation maneuvers (neurologists = 24%, ENT physicians = 33%, PCPs = 50%) and reposition maneuvers (28, 61, and 84%) for posterior canal BPPV in substantial fractions of participants (21), we found substantially higher rates of use of provocation and repositioning maneuvers in both PCPs and specialists working in Switzerland. These significant national differences emphasize the importance to adapt teaching activities to the specific needs of a given country.

Unlike PCPs' (3), specialists rarely prescribe anti-vertiginous drugs or antiemetic drugs to patients with suspected BPPV, being in line with best practice (22, 23). Noteworthy, over 90% of specialists interviewed indicated the frequent prescription of physical therapy in patients with episodic or chronic dizziness, emphasizing the popularity of non-pharmaceutical treatment strategies in this setting. Compared to ENT physicians, the fraction of neurologists that would always prescribe physical therapy in patients with episodic/chronic dizziness was twice as large, whereas ENT physicians prescribed anti-vertiginous drugs significantly more often (40 vs. 5%). With antiemetics and anti-vertiginous drugs being primarily used as intermittent, acute-phase treatment, their value in episodic or chronic conditions is limited, and thus their use should be critically reviewed and preferentially used for treating periods with symptom exacerbation only. Overall, these findings indicate substantial differences in prescription behavior depending on the training background. Noteworthy, a patient selection bias may result in distinct patient populations presenting preferentially to either neurologists or ENT physicians, resulting in diverging treatment strategies.

### 4.4 Study limitations

A number of limitations should be kept in mind. First, participation in this online survey was by invitation and thus not mandatory. This may have caused a selection bias; specifically, those specialists being most interested in taking care of the dizzy patient may have been overrepresented, and those specialists with a high workload may have been more likely not to participate. With about 900 registered board-certified ENT physicians and about 1,000 neurologists working in Switzerland (based on the listing of the Swiss Medical Association), our sample of 111 completed questionnaires represents only about 5% of all specialists. Second, we collected data on the specialists' self-reported diagnostic and therapeutic procedures. Thus, in practice, specialists may diverge from these procedures for a specific patient. Third, we cannot exclude a recall bias, resulting in either too high or too low numbers with regard to specific diagnoses made or tests applied. Fourth, the two specialties (neurology and ENT) participating were distinct both with regard to the age distribution (with neurologists being younger on average), professional experience (being larger in the ENT physicians), and working place (with more neurologists working in hospitals). Fifth, based on an initial triage by the referring physician, neurologists, and ENT physicians likely will deal with—at least partially—distinct patient populations. Thus, differences in the diagnostic tests ordered and treatments initiated between neurologists and ENT physicians (such as ordering MRI or prescribing anti-vertiginous drugs) could well be explained by distinct underlying disorders. Importantly, we fell short of the targeted sample size (75 participants for each of both specialties), reaching only a total of 111 completed questionnaires, and specialists from the Latin part of Switzerland were relatively underrepresented (making up only 22% of all participants).

## 5 Conclusion

We noted substantial differences in both the diagnostic workup and the treatment approaches for the dizzy patient, depending on the training background of the specialists involved. Thus, promoting knowledge to neurologists and ENT physicians should be stratified for training background. Specifically, training for lateral canal BPPV repositioning maneuvers and testing for hearing loss should be prioritized by neurologists. Furthermore, neurologists tended to prescribe physical therapy more often and anti-vertiginous drugs less often, and to order MRI in acutely dizzy patients more frequently. Awareness for preferential use of non-pharmacological treatments in acutely dizzy patients (except steroids in acute peripheral vestibulopathy) should be increased, especially among ENT physicians. Among the key examination techniques recommended to assess acutely dizzy patients, testing for skew deviation was the least popular and should be emphasized more in the continuous education of specialists.

## Data availability statement

The raw data supporting the conclusions of this article will be made available by the authors, without undue reservation.

## Author contributions

AZ: Conceptualization, Formal analysis, Methodology, Validation, Writing – review & editing. GM: Conceptualization, Formal analysis, Methodology, Validation, Writing – review & editing. DH: Formal analysis, Methodology, Writing – review & editing. HK: Conceptualization, Formal analysis, Methodology, Writing – review & editing. SD: Formal analysis, Methodology, Writing – review & editing. RK: Formal analysis, Methodology, Writing – review & editing. AK: Formal analysis, Methodology, Writing – review & editing. CC: Formal analysis, Methodology, Writing – review & editing. AW-L: Formal analysis, Methodology, Writing – review & editing. AT: Conceptualization, Data curation, Formal analysis, Investigation, Methodology, Project administration, Resources, Supervision, Validation, Visualization, Writing – original draft, Writing – review & editing.
