# Supplementary material for: What is the current status of care by neuro-otology specialists in Switzerland—A national survey
Source: Front Neurol. 2023 Dec 7;14:1322330. doi: 10.3389/fneur.2023.1322330 (PMC10733682; doi:10.3389/fneur.2023.1322330)
Supplement: Supplementary file 1 [file Data_Sheet_1.PDF]

What is the current status of care by neuro-otology specialists in Switzerland – a national survey

Supplementary material

Table S1: Top diagnoses in dizzy patients – comparison between ENT physicians and neurologists

**Table S1: Top diagnoses in dizzy patients – comparison between ENT physicians and neurologists**	
**Diagnoses made (in order of *decreasing* frequency)**	
**ENT physicians**	**Neurologists**
1. Benign paroxysmal positional vertigo (BPPV)	1. Benign paroxysmal positional vertigo (BPPV)
2. Acute unilateral vestibulopathy*	2. Multifactorial dizziness
2. Multifactorial dizziness*	3. Dizziness / gait imbalance linked to peripheral polyneuropathy*
4. Functional dizziness (“phobic vertigo”)*	3. Functional dizziness (“phobic vertigo”)*
4. Menière’s disease*	5. Acute unilateral vestibulopathy
6. Vestibular migraine	6. Vestibular migraine
7. Vertigo or dizziness of unclear origin	7. Vertigo or dizziness of unclear origin
8. Dizziness / gait imbalance linked to peripheral polyneuropathy	8. Menière’s disease
9. Vertigo or dizziness related to cardiovascular disease	9. Vertigo or dizziness related to cardiovascular disease

* These diagnoses were equally frequently made by either ENT physicians or neurologists.

Table S2: Predictors for ordering brain MRI in patients with suspected acute unilateral vestibulopathy

Table S2: Predictors for ordering brain MRI in patients with suspected acute unilateral vestibulopathy			
	**Univariable**		
**Predictor**	**Sample size (n)**	**Odds ratio (95% CI)**	**p-value**
Age			0.567
Aged 30-40 years	29	0.93 (0.32-2.67)	0.893
Aged 41-50 years	33	1.59 (0.55-4.60)	0.391
Aged 51-60 years	28	0.86 (0.30-2.47)	0.775
Aged > 60 years	21	[Ref.]	
Gender			
Male	71	[Ref.]	
Female	40	1.64 (0.80-3.37)	0.177
Years of professional experience	111	0.97 (0.70-1.35)	0.854
Location of the specialist's working place			
German part of Switzerland	87	[Ref.]	
Latin part of Switzerland	24	1.41 (0.63-3.18)	0.401
Location of specialist's working place			
Hospital (academic/non-academic)	49	[Ref.]	
Private practice	62	0.70 (0.35-1.39)	0.305
Specialty			
Neurology	62	[Ref.]	
ENT	49	0.33 (0.16-0.67)	0.002
Number of dizzy patients seen monthly	111	0.99 (0.97-1.01)	0.338
Score "oculomotor and vestibular signs" (0-100%)*	110	0.98 (0.69-1.41)	0.925
	**Multivariable**		
**Predictor**	**Sample size (n)**	**Adjusted odds ratio (95% CI)**	**p-value**
Specialty			
Neurology	62	[Ref.]	
ENT	49	0.33 (0.16-0.67)	0.002

Abbreviations: CI=confidence interval; ENT=ear-nose-throat; Ref=reference category. Logistic regressions were used.

* The score "subtle oculomotor and vestibular signs" combined testing for the head-impulse test, for the presence of a gaze-evoked nystagmus and a skew deviation and for spontaneous nystagmus with fixation preserved and with fixation removed.

Table S3: Predictors of therapies prescribed by the specialists (n=111)

Table S3: Predictors of therapies prescribed by the specialists*			
**Treatment initiated – acute vertigo / dizziness**	**Response variable (per 10%)**	**odds ratio (OR) (95% CI)**	**p-value**
Physical therapy	Fraction of cases with unclear diagnosis after initial assessment	1.10 (0.94-1.29)	0.232
Physical therapy	Number of dizzy patients seen per month	1.01 (1.00-1.03)	0.054
Antiemetics	Fraction of cases with unclear diagnosis after initial assessment	1.00 (0.87-1.15)	0.983
Antiemetics	Number of dizzy patients seen per month	1.01 (0.99-1.03)	0.254
Anti-vertiginous drugs	Fraction of cases with unclear diagnosis after initial assessment	0.89 (0.76-1.04)	0.145
Anti-vertiginous drugs	Number of dizzy patients seen per month	1.00 (0.98-1.02)	0.928
**Treatment initiated – episodic / chronic vertigo / dizziness**	**Response variable (per 10%)**	**odds ratio (OR) (95% CI)**	**p-value**
Physical therapy	Fraction of cases with unclear diagnosis after initial assessment	1.04 (0.94-1.16)	0.418
Physical therapy	Number of dizzy patients seen per month	1.01 (0.99-1.02)	0.278
Antiemetics	Fraction of cases with unclear diagnosis after initial assessment	1.15 (0.99-1.34)	0.065
Antiemetics	Number of dizzy patients seen per month	1.00 (0.98-1.02)	0.970
Anti-vertiginous drugs	Fraction of cases with unclear diagnosis after initial assessment	1.08 (0.96-1.21)	0.214
Anti-vertiginous drugs	Number of dizzy patients seen per month	0.99 (0.97-1.01)	0.264

* Fractional logistic regressions were used. Odds ratios (ORs) are provided to indicate if the likelihood of initiating a given treatment was correlated with a given response variable (e.g. if it became significantly more likely to prescribe physical therapy with an increasing fraction of cases with unclear diagnosis). ORs are considered significant if the 95% confidence interval (CI) does not include 1.0 and if the p-value is <0.05.

Abbreviations: CI=confidence interval.

Specialist Questionnaire:

3 sections

1. **Status Quo Regarding Diagnosis/Treatment**
2. **Current Problems in the Treatment of Patients with Dizziness by Specialists with a Focus on Specialists in Neurology and ENT**
3. **Prospects for the Future—Desired Improvements**

Please note that only health care professionals are authorized to complete this questionnaire. Compensation will only be provided if this condition applies and if you have completed the questionnaire in full.

Status Quo Regarding Diagnosis/Treatment

Key Epidemiological Data

* 1. How old are you?

- ○ <30 years
- ○ 30–40 years
- ○ 41–50 years
- ○ 51–60 years
- ○ >60 years

* 2. What is your gender?

- ○ male
- ○ female
- ○ not specified

* 3. How many years of professional experience do you have (since completing your medical training)?

0 years 40

* 4. Which medical specialties are you certified in?

- ☐ Allergies
- ☐ Surgery
- ☐ Dermatology
- ☐ FMH [Swiss Medical Association]-certified general internal medicine
- ☐ Gastroenterology
- ☐ Hematology
- ☐ Cardiology
- ☐ Nephrology
- ☐ Neurosurgery
- ☐ Neurology
- ☐ ENT
- ☐ Pediatrics
- ☐ Pulmonology
- ☐ Psychiatry
- ☐ Urology
- ☐ Other

* 5. How many physicians work in your practice?

- ○ Solo practice
- ○ 2–4
- ○ 5–8
- ○ >8

* 6. On average, how many patients do you see per day?

0 patients 50

* 7. On average, how much time do you spend with each patient?

0 minutes 30

* 8. Please describe the location of your clinical practice

- ○ City practice
- ○ Practice is in a greater metropolitan area
- ○ Country practice
- ○ University hospital
- ○ Non-university hospital

* 9. In which Canton of Switzerland is your clinical practice located?

○ Aargau (AG)
○ Appenzell Ausserrhoden (AR)
○ Appenzell Innerrhoden (AI)
○ Basel-City (BS)
○ Basel-Country (BL)
○ Bern (BE)
○ Freiburg (FR)
○ Geneva (GE)
○ Glarus (GL)
○ Graubünden (GR)
○ Jura (JU)
○ Lucerne (LU)
○ Neuchâtel (NE)
○ Nidwalden (NW)
○ Obwalden (OW)
○ St. Gallen (SG)
○ Schaffhausen (SH)
○ Schwyz (SZ)
○ Solothurn (SO)
○ Thurgau (TG)
○ Ticino (TI)
○ Uri (UR)
○ Valais (VS)
○ Vaud (VD)
○ Zug (ZG)
○ Zurich (ZH)

Status Quo Regarding Diagnosis/Treatment

Existing Treatment of Patients with Dizziness

* 10. How many patients with a cardinal symptom of dizziness do you see per month?

0 percent (%) 100

* 11. Of these, what proportion are patients with acute dizziness symptoms?

0 percent (%) 100

* 12. Of these, what proportion are patients with episodic dizziness symptoms (in the form of attacks)?

0 percent (%) 100

* 13. Of these, what proportion are patients with chronic (persistent) dizziness?

0 percent (%) 100

* 14. On average, how much time do you spend on each patient with dizziness?

- ○ Less time than for normal patients
- ○ Same time as for normal patients
- ○ More time than for normal patients

* 15. What questions do you consider particularly important in diagnostic terms when taking the history of patients presenting with dizziness as a cardinal symptom?

	Not applicable at all	Somewhat inapplicable	Somewhat applicable	Definitely applicable	No answer
What type of dizziness occurred (vertigo, feeling off-balance, lightheadedness, unsteady gait)?	◯	◯	◯	◯	◯
How often do the attacks of dizziness occur?	◯	◯	◯	◯	◯
Is the dizziness triggered by certain movements?	◯	◯	◯	◯	◯
Is the dizziness triggered in certain situations?	◯	◯	◯	◯	◯
How long does an attack of dizziness last?	◯	◯	◯	◯	◯
How intense is the dizziness?	◯	◯	◯	◯	◯
Is the dizziness accompanied by nausea and vomiting?	◯	◯	◯	◯	◯
Does the patient feel a tendency to fall in one direction?	◯	◯	◯	◯	◯
Are there any ear disorders present (hearing loss, tinnitus, ear pain)?	◯	◯	◯	◯	◯
Is the dizziness accompanied by other symptoms?	◯	◯	◯	◯	◯
Prior/current medication history	◯	◯	◯	◯	◯
Did the patient experience trauma beforehand (cranial, cervical spine)?	◯	◯	◯	◯	◯

* 16. How important do you consider the following tests to be for patients presenting with dizziness as a cardinal symptom in your clinical practice?

	Not important at all	Somewhat unimportant	Somewhat important	Very important	No answer
Gait tests (tandem gait test, tandem gait test with eyes closed)	◯	◯	◯	◯	◯
Romberg test	◯	◯	◯	◯	◯
Unterberger’s test	◯	◯	◯	◯	◯
Assessment for spontaneous nystagmus with fixation	◯	◯	◯	◯	◯
Assessment for spontaneous nystagmus with suppressed fixation (e.g. using Frenzel goggles)	◯	◯	◯	◯	◯
Head impulse test	◯	◯	◯	◯	◯
Assessment for gaze-evoked nystagmus	◯	◯	◯	◯	◯
Alternate cover test	◯	◯	◯	◯	◯
Search for hearing loss (rubbed fingers, quiet speech)	◯	◯	◯	◯	◯
Positional maneuvers for suspected benign paroxysmal positional vertigo (BPPV)	◯	◯	◯	◯	◯
General neurological examination (e.g. paralysis, dysesthesia)	◯	◯	◯	◯	◯
Checking ocular motor function (evidence of ocular palsy?)	◯	◯	◯	◯	◯
Otoscopy	◯	◯	◯	◯	◯

* 17. Which diagnostic maneuvers for suspected BPPV are you familiar with?

- ◻ Dix-Hallpike maneuver
- ◻ Supine roll test/barbecue 90°
- ◻ Inverse Hallpike maneuver
- ◻ Bow and lean test

* 18. Which diagnostic maneuvers are used in your practice?

- ☐ Dix-Hallpike maneuver
- ☐ Supine roll test/barbecue 90°
- ☐ Inverse Hallpike maneuver
- ☐ Bow and lean test

* 19. Which of the following testing instruments are available in your clinical practice for you to use?

- ☐ Frenzel goggles
- ☐ Video Frenzel goggles
- ☐ Otoscope
- ☐ Eye chart
- ☐ Hearing test (including smartphone-based hearing tests)
- ☐ Tuning fork for vibration testing (for polyneuropathy)
- ☐ Video head impulse test
- ☐ Video oculography (ocular motor function)
- ☐ Caloric analysis
- ☐ Ocular vestibular-evoked myogenic potential (oVEMP)
- ☐ Cervical vestibular-evoked myogenic potential (cVEMP)
- ☐ Subjective visual vertical
- ☐ Platform posturography
- ☐ Multiaxial chair for positioning/treatment of patients with suspected BPPV
- ☐ Multiaxial chair for testing the optokinetic reflex and vestibulo-ocular reflex.
- ☐ None of the above

* 20. What proportion of patients with acute dizziness do you refer to a specialist in another discipline for further diagnostic workup?

0 percent (%) 100

* 21. What proportion of patients with chronic/episodic dizziness do you refer to a specialist in another discipline for further diagnostic workup?

0 percent (%) 100

* 22. What proportion of patients with acute dizziness do you refer to the specialist outpatient dizziness clinic/a dizziness center for further diagnostic workup?

0 percent (%) 100

* 23. What proportion of patients with chronic/episodic dizziness do you refer to the specialist outpatient dizziness clinic/a dizziness center for further diagnostic workup?

0 percent (%) 100

* 24. From which specialties do you most often get patients with dizziness? Please sort the following answers by clicking, dragging, and dropping them in the right order.

- Neurology
- ENT
- Emergency
- Interdisciplinary dizziness clinic
- Cardiology
- Psychiatry
- Neurosurgery
- Spinal surgery

* 25. What are your most common diagnoses for patients presenting with dizziness as a cardinal symptom? Please sort the following answers by clicking, dragging, and dropping them in the right order (top 7, N/A: not applicable).

≣ ▭	BPPV (benign paroxysmal positional vertigo)	☐ N/A
≣ ▭	Somatoform dizziness (phobic vertigo)	☐ N/A
≣ ▭	Vestibular neuritis	☐ N/A
≣ ▭	Dizziness/unsteady gait associated with polyneuropathy	☐ N/A
≣ ▭	Multifactorial dizziness	☐ N/A
≣ ▭	Dizziness of unknown etiology	☐ N/A
≣ ▭	Vestibular migraine	☐ N/A
≣ ▭	Meniere’s disease	☐ N/A
≣ ▭	Cardiovascular causes	☐ N/A
≣ ▭	Other cardiovascular causes	☐ N/A

* 26. In your opinion, which findings in patients with acute dizziness require immediate further investigation/diagnostic workup?

	Not applicable at all	Rarely applicable	Frequently applicable	Always applicable	No answer
Markedly unsteady gait	◯	◯	◯	◯	◯
Nausea and vomiting	◯	◯	◯	◯	◯
Concomitant paralysis, dysarthria, dysesthesia, or vision problems	◯	◯	◯	◯	◯
Presence of nystagmus	◯	◯	◯	◯	◯
Accompanying unilateral, newly occurring hearing loss	◯	◯	◯	◯	◯
A tendency to fall when sitting unsupported or standing unassisted, requiring the patient to be caught.	◯	◯	◯	◯	◯
Isolated headaches	◯	◯	◯	◯	◯
Isolated tinnitus	◯	◯	◯	◯	◯
Elevated blood pressure	◯	◯	◯	◯	◯

Status Quo Regarding Diagnosis/Treatment

Therapeutic Measures

* 27. To what proportion of your patients presenting with acute dizziness as a cardinal symptom do you prescribe targeted physical therapy (balance training)?

0 percent (%) 100

* 28. To what proportion of your patients presenting with chronic/episodic dizziness as a cardinal symptom do you prescribe targeted physical therapy (balance training)?

0 percent (%) 100

* 29. To what proportion of your patients presenting with acute dizziness as a cardinal symptom do you prescribe antiemetics?

0 percent (%) 100

* 30. To what proportion of your patients presenting with chronic/episodic dizziness as a cardinal symptom do you prescribe antiemetics?

0 percent (%) 100

* 31. To what proportion of your patients presenting with acute dizziness as a cardinal symptom do you prescribe antivertigo medications?

0 percent (%) 100

* 32. To what proportion of your patients presenting with chronic/episodic dizziness as a cardinal symptom do you prescribe antivertigo medications?

0 percent (%) 100

* 33. Which antivertigo medications do you regularly prescribe?

- ☐ Betahistine
- ☐ Ginkgo biloba extracts
- ☐ Corticosteroids
- ☐ Flunarizine
- ☐ Cinnarizine + dimenhydrinate
- ☐ Other

* 34. In your opinion, which of the following statements apply for patients with suspected benign paroxysmal positional vertigo (BPPV)?

	Not applicable at all	Rarely applicable	Frequently applicable	Always applicable	No answer
I prescribe antivertigo medications for patients with suspected BPPV	◯	◯	◯	◯	◯
I prescribe antiemetics for patients with suspected BPPV	◯	◯	◯	◯	◯
I perform provocation maneuvers for patients with suspected BPPV	◯	◯	◯	◯	◯
I provide self-repositioning instructions to patients with suspected BPPV	◯	◯	◯	◯	◯
I provide a brochure or outline as self-repositioning instructional material to patients with suspected BPPV	◯	◯	◯	◯	◯
I provide patients with suspected BPPV with links to online videos as self-repositioning instructional material	◯	◯	◯	◯	◯
I prescribe vitamin D to patients with recurrent BPPV	◯	◯	◯	◯	◯

* 35. Which of the following repositioning maneuvers do you perform with patients who have confirmed BPPV?

- ☐ Epley maneuver
- ☐ Semont maneuver
- ☐ Gufoni maneuver
- ☐ Barbecue maneuver
- ☐ Other

* 36. Which of the following steps do you take if you diagnose a patient with suspected acute vestibular neuritis (i.e. acute inflammation of the vestibular nerve)?

	Not applicable at all	Rarely applicable	Frequently applicable	Always applicable	No answer
Referral to other specialists (neurology/ENT)	○	○	○	○	○
Referral to the Emergency Room	○	○	○	○	○
Referral to Radiology for a cranial CT	○	○	○	○	○
Referral to Radiology for a cranial MRI	○	○	○	○	○
Treatment with steroids	○	○	○	○	○
Treatment with virostatics	○	○	○	○	○
Treatment with antiemetics	○	○	○	○	○
Treatment with antivertigo medications	○	○	○	○	○

* 37. Which of the following steps do you take for patients with chronic/episodic dizziness (lasting >3 months)?

	Not applicable at all	Rarely applicable	Frequently applicable	Always applicable	No answer
Referral to other specialists (neurology/ENT)	◯	◯	◯	◯	◯
Referral to an interdisciplinary dizziness clinic	◯	◯	◯	◯	◯
Performance of provocation maneuvers	◯	◯	◯	◯	◯
Treatment with antivertigo medications	◯	◯	◯	◯	◯
Treatment with antiemetics	◯	◯	◯	◯	◯
Treatment with physical therapy (focused on balance training/gait training)	◯	◯	◯	◯	◯
None, you wait and see	◯	◯	◯	◯	◯

Limitations in Diagnosing/Treating Patients Presenting with Dizziness as a Cardinal Symptom

* 38. For your patients presenting with acute dizziness as a cardinal symptom, how often does the diagnosis remain unclear after the initial consultation?

0 percent (%) 100

* 39. For your patients presenting with chronic/episodic dizziness as a cardinal symptom, how often does the diagnosis remain unclear after the initial consultation?

0 percent (%) 100

* 40. For your patients presenting with acute dizziness as a cardinal symptom, how often does the diagnosis remain unclear even after further diagnostic workup arranged by you?

0 percent (%) 100

* 41. For your patients presenting with chronic/episodic dizziness as a cardinal symptom, how often does the diagnosis remain unclear even after further diagnostic workup arranged by you?

0 percent (%) 100

* 42. Do you feel equipped to perform the diagnostic workup for patients presenting with acute dizziness as a cardinal symptom?

- ○ Not applicable at all
- ○ Rarely applicable
- ○ Frequently applicable
- ○ Always applicable
- ○ No answer

* 43. Do you feel equipped to perform the diagnostic workup for patients presenting with chronic/episodic dizziness as a cardinal symptom?

- ○ Not applicable at all
- ○ Rarely applicable
- ○ Frequently applicable
- ○ Always applicable
- ○ No answer

* 44. Are you satisfied with the results of the diagnostic workup initiated for patients presenting with acute dizziness as a cardinal symptom?

- ○ Not applicable at all
- ○ Rarely applicable
- ○ Frequently applicable
- ○ Always applicable
- ○ No answer

* 45. Are you satisfied with the results of the diagnostic workup initiated for patients presenting with chronic/episodic dizziness as a cardinal symptom?

- ○ Not applicable at all
- ○ Rarely applicable
- ○ Frequently applicable
- ○ Always applicable
- ○ No answer

* 46. Do you feel equipped to treat patients presenting with acute dizziness as a cardinal symptom?

- ○ Not applicable at all
- ○ Rarely applicable
- ○ Frequently applicable
- ○ Always applicable
- ○ No answer

* 47. Do you feel equipped to treat patients presenting with chronic/episodic dizziness as a cardinal symptom?

- ○ Not applicable at all
- ○ Rarely applicable
- ○ Frequently applicable
- ○ Always applicable
- ○ No answer

Prospects for the Future—Desired Improvements

* 48. What would you like to see from the referring physician to improve the care of patients presenting with dizziness as a cardinal symptom?

	Not applicable at all	Rarely applicable	Frequently applicable	Always applicable	No answer
Improvement of the dialog between specialists and primary care providers	○	○	○	○	○
Faster referral to the specialist for unclear situations	○	○	○	○	○
Selective referral of patients to the specialist	○	○	○	○	○
More specific information about the referral and diagnostic workup/treatments performed to date	○	○	○	○	○
More specific information about the urgency of the specialist’s evaluation	○	○	○	○	○
Consistent re-acceptance of patients by referring physicians	○	○	○	○	○

* 49. Which of the following are appropriate steps to help you improve your familiarity with issues related to dizziness as a specialist?

	Not applicable at all	Rarely applicable	Frequently applicable	Always applicable	No answer
Webinars (digital)	○	○	○	○	○
Hands-on courses/workshops (in-person)	○	○	○	○	○
National recommendations, guidance paper (print)	○	○	○	○	○
Practice recommendations (print)	○	○	○	○	○
Smartphone apps to convey information or for recommendations (digital)	○	○	○	○	○

* 50. Which of the following tools for specialists would be helpful for diagnosis?

- ☐ Web-based diagnostic pathway/algorithm (digital)
- ☐ App for diagnostic pathway/algorithm (digital)
- ☐ Web-portal with clinical pictures and sample case reports (digital)
- ☐ Other

* 51. Which of the following tools for specialists would be helpful for treatment?

- ☐ Web-based therapeutic pathway (digital)
- ☐ Web-portal with clinical pictures and sample case reports (digital)
- ☐ App for therapeutic pathway (digital)
- ☐ Other

* 52. Which of the following tools for specialists would be helpful for follow-up?

- ☐ Web-based follow-up (digital)
- ☐ App for follow-up with dizziness diary (digital)
- ☐ Dizziness diary (print)
- ☐ Web-portal with clinical pictures and sample case reports (digital)
- ☐ Other

* 53. Which of the following tools for patients would be helpful for patient education & treatment management?

- ☐ Web-based platform (digital)
- ☐ App-based platform (digital)
- ☐ Brochure for patients (print)
- ☐ Dizziness diary (print)
- ☐ Flyer for patients (print)
- ☐ Other

Personal Data:

All personal information on this site is solely for the purpose of providing the participant with compensation for completing the questionnaire. All personal information will be treated in confidence and will neither be divulged to third parties, nor saved, nor used for statistical purposes.

* 54. Personal data

Title

First name

Last name

Street/number

ZIP code/City

Bank details

GLN number

55. Optional information

Hospital

Department

* 56. I hereby confirm that I am a health care professional.

- ☐ Yes
